# Recanalization and reperfusion in clinically-relevant porcine model of stroke

**DOI:** 10.3389/fnins.2025.1572925

**Published:** 2025-06-05

**Authors:** Błażej Nowak, Piotr Holak, Izabela Małysz-Cymborska, Alexandra Chovsepian, Yanina Dening, Jarosław Olszewski, Aleksandra Piecuch, Maria Jasieniak, Jakub Jasieniak, Arkadiusz Szterk, Maria Sady, Karolina Ferenc, Daniel Berchtold, Artur Jabłoński, Romuald Zabielski, Zdzisław Gajewski, Tim Magnus, Mirosław Janowski, Piotr Walczak, Andreas Meisel, Francisco Pan-Montojo, Dominika Gołubczyk

**Affiliations:** ^1^Division of Interventional Neuroradiology, Department of Radiology, The National Medical Institute of the Ministry of the Interior and Administration, Warsaw, Poland; ^2^Department of Surgery and Roentgenology With a Clinic, Faculty of Veterinary Medicine, University of Warmia and Mazury in Olsztyn, Olsztyn, Poland; ^3^Department of Neurosurgery, Collegium Medicum, University of Warmia and Mazury in Olsztyn, Olsztyn, Poland; ^4^NEUREVO GmbH, Munich, Germany; ^5^Center for Translational Medicine, Warsaw University of Life Sciences, Warsaw, Poland; ^6^Department of Neurology with Experimental Neurology, Charité - Universitätsmedizin Berlin, Corporate Member of Freie Universität Berlin and Humboldt-Universität Zu Berlin, Berlin, Germany; ^7^Department of Pathology and Veterinary Diagnostics, Warsaw University of Life Sciences, Warsaw, Poland; ^8^Department of Neurology, University Medical Center Hamburg-Eppendorf, Hamburg, Germany; ^9^Program in Image Guided Neurointerventions, Department of Diagnostic Radiology and Nuclear Medicine, University of Maryland, Baltimore, MD, United States; ^10^Department of Neurology, Neurological Clinic am Sorpesee, Sundern, Germany; ^11^IIS Biogipuzkoa, Donostia University Hospital, San Sebastian, Spain; ^12^Department of Psychiatry and Psychotherapy, Ludwig-Maximilian University Hospital, Munich, Germany; ^13^Ti-com LLC, Olsztyn, Poland

**Keywords:** acute stroke therapy, ischaemic stroke, MRI, radiology, reperfusion, rtPA, stroke, thrombolysis

## Abstract

**Introduction:**

Stroke is a leading cause of death and long-term disability. Pigs have been considered an ideal large animal model in biomedicine; however, the complex vascular anatomy has posed challenges for stroke research. Nonetheless, we have previously overcome these limitations and demonstrated the feasibility of endovascularly inducing stroke in pigs. Here, we study to further mimic clinical situation by achieving recanalization, which has not been previously accomplished.

**Methods:**

A stroke was induced in eight juvenile male domestic pigs. In anaestethised animals catheter was placed in the ascending pharyngeal artery near the rete mirabile (RM) under X-ray guidance. The animals were then transferred to an MRI scanner. Gadolinium-based contrast agent (GBCA) was infused at various speeds until transcatheter cerebral perfusion was visible on MRI. Subsequently, a mixture of thrombin and GBCA was infused, and the retention of contrast on MRI scans proved successful induction of thrombosis. Subsequent DWI and PWI MR images confirmed the successful induction of stroke. Two hours after ischemia, we intra-arterially infused rtPA (20 mg) and confirmed recanalization of the thrombosed vessels using MRI. One month later the stroke was confirmed through follow-up MRI scans and post-mortem histological and immunohistochemical analyses.

**Results:**

We successfully induced stroke with an average lesion size based on ADC at 8.18 ± 4.98 cm^3^, ranging from 3.27 to 17.33 cm^3^. After recanalization, the severely hypoperfused area (Tmax>6) was only 1.168 ± 0.223 cm^3^. Subsequent histological analysis revealed neuronal loss within the lesion, the formation of astrocytic scar tissue, and elevated levels of activated microglia.

**Discussion:**

Our study demonstrates the successful recanalization of cerebral vasculature in porcine model of ischemic stroke. It makes the model highly relevant to the current clinical workflow and offers an attractive avenue for studying novel diagnostics, therapeutics and further exploration of the underlying pathomechanisms. The feasibility of continuous MR imaging throughout the entire procedure facilitates the achievement of the aforementioned goals more readily.

## Introduction

Stroke research has gained significant interest over the past two decades due to its significant clinical importance and the success of some therapeutic interventions. Despite these efforts, stroke remains the second-leading cause of death and the third-leading cause of death and disability combined (GBD 2019 Stroke Collaborators, 2021). The primary therapeutic approach for acute ischemic stroke (AIS) involves rapid restoration of cerebral blood flow, with early intervention leading to significant improvement, while neuroprotection-based strategies have been less successful. Reperfusion therapy aims to improve clinical outcomes by restoring anterograde perfusion on obstructed vessels and salvaging ischemic brain tissue. Early recanalization is strongly associated with improved functional outcomes and reduced mortality (Marks et al., 2014). Two approved therapeutic reperfusion strategies are intravenous thrombolysis with recombinant tissue plasminogen activator (tPA) and endovascular mechanical thrombectomy (EMT). The recanalization rate is 46% for intravenous thrombolysis and up to 84% for mechanical thrombectomy, resulting in good outcomes in 58.1% of recanalized patients vs. 24.8% of non-recanalized patients (Rha and Saver, 2007). There is an additional effort to supplement thrombectomy with intra-arterial tPa administration (Renú et al., 2022). However, these approaches have limitations, such as a short therapeutic window (<4.5 h) for rtPA and up to 24 h for mechanical thrombectomy in highly selected patients (such as large vessel occlusion or DWI-FLAIR mismatch), leading to only 25% of patients receiving rtPA and 18% receiving mechanical thrombectomy in well-organized stroke centers. It means that most AIS patients receive only supportive treatment, driving research into developing new therapeutic avenues to broaden the inclusion criteria for current interventions and identify neuroprotective strategies to prevent irreversible brain damage (Walczak et al., 2024). More than 1,000 neuroprotective agents have been shown to be effective in small animal studies but have failed in human clinical trials (Savitz et al., 2011). These failures underscore the poor clinical relevance of rodent models and the uncertainty surrounding the brain accumulation of systemically injected drugs. Our group has demonstrated that brain accumulation of systemically injected biologics is minimal (Lesniak et al., 2019a,b), highlighting the need for animal models with improved clinical relevance to be integrated into translational pipelines, including potential benefits for intra-arterial drug deliveries. Given the comparable cerebrovasculature to humans, pig stroke models may have utility in bridging the gap between small animal models and clinical evaluation and drive enhanced clinical translation.

Compared to the lissencephalic brains of rodents, pigs have highly gyrencephalic brains that resemble humans. They have lobes, gyri, sulci, fissures, and organization of motor and somatosensory areas comparable to higher mammals. Additionally, pigs have a white-to-gray matter ratio and neurovascular characteristics such as cerebral vessel diameter similar to those of humans. These factors may be critical for improving translational success from small animal studies, which have had limited success in human clinical trials. There are currently five reported approaches to inducing focal brain ischemia in swine, including electrocoagulation (Zhang et al., 2007; Knight, 2000; Sakoh et al., 2000, 2001; Røhl et al., 2002; Watanabe et al., 2007), clip/ligature occlusion (Sakoh et al., 2001; Schöll et al., 2017), endovascular embolization (Mangla et al., 2015), photothrombosis (Kuluz et al., 2007; Armstead et al., 2010, 2012, 2016), and endothelin-1 (ET-1) injection (Zhang et al., 2016; d'Esterre et al., 2015; Elliott et al., 2014; Wright et al., 2016). However, none of these methods allow for pharmacological reperfusion (Li et al., 2022), which is essential to make animal models relevant to clinical practice in the mechanical or pharmacological thrombolysis era.

We previously reported the feasibility of inducing endovascular, thrombin-induced ischemic stroke in a swine model under real-time MRI (Golubczyk et al., 2020). This model offers significant advantages, such as a minimally invasive approach without any surgery-related morbidity and the ability to monitor stroke evolution in real-time using MRI. In the current study, we have further developed this model and demonstrated the feasibility of pharmacological thrombolysis using intra-arterial rtPA.

## Materials and methods

All animal procedures were approved by the local Ethics Committee of the Warsaw University of Life Sciences in Warsaw, Poland (WAW2/046/2021).

### *In vitro* tPA thrombolytic activity assay with a porcine blood clot

Venous blood (3 ml) was collected from five healthy male domestic pigs, and measurements were performed in triplicates using fresh blood. The blood was placed into 5 ml tubes and incubated for 2 h for clot formation in room temperature without adding of any additional medium. Clots were incubated in a plasma medium to maintain plasminogen activity. The thrombolytic activity of alteplase was tested in two different settings: (i) with natively coagulated blood and (ii) in blood pre-treated with thrombin on orbital shaker (50 RPM). For the native clot assay, five distinct conditions were tested: (1) without tPA treatment, (2) with a high single dose of tPA (0.1 mg/ml), (3) with a high dose of tPA (0.1 mg/ml) added every hour, five times in total, (4) with a low single dose of tPA (0.01 mg/ml), and (5) with a low dose of tPA added (0.01 mg/ml) every hour, five times in total. Doses of tPA added to vials was chosed based on publication of Frenkel et al. (2006). In addition, the clot weight was measured hourly for 10 h after blood collection. Both assays were performed at room temperature.

### Stroke induction experiments

**Figure 1:**
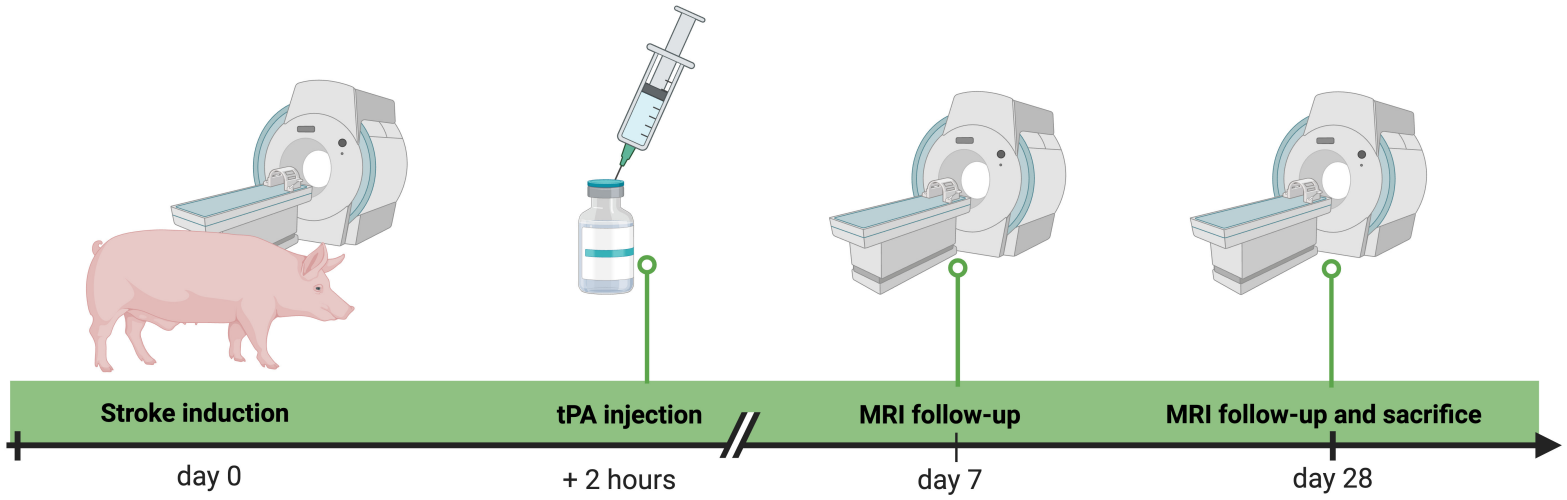
The experimental outline of the *in vivo* study.

A schematic representation of the *in vivo* experimental design is shown in Figure 1. Eight male pigs (Polish white lop-eared) weighting an average of 33.95 ± 5.15 kg obtained from a local farmer were used in this study. The protocol was conducted in compliance with the European Union's regulations concerning the protection of experimental animals. All animal experiments were performed in accordance with the approved protocol and ARRIVE guidelines. The stroke induction treatments were performed as described in our previous reports (Golubczyk et al., 2020; Chovsepian et al., 2022). Initially, the animals were anesthetized using atropine (0.05 mg/kg i.m.), xylazine (3 mg/kg i.m.), and ketamine (6 mg/kg i.m.). Before intubation, the animals were given propofol (5 mg/kg/h i.v.), and after intubation, the anesthesia was maintained using isoflurane (1–3%). A sterile surgical field was prepared, and a percutaneous 4F sheath was inserted into the pig's right femoral artery. Using the C-arm for guidance, the 4F vertebral catheter (Balton, Poland) was advanced through the aortic arch, into the left carotid artery, and then into the ascending pharyngeal artery. The catheter was then secured in the ascending pharyngeal artery, ~1–2 mm away from the rete mirabile. The animals were subsequently transferred to a 3 Tesla magnetic resonance imaging (MRI) scanner (GE Healthcare).

Baseline MRI scans were obtained, and the animals' blood pressure was lowered to a target systolic pressure of approximately 60 mmHg using sodium nitroprusside (0.2–0.50 mg/kg/min). At this point, a thrombin solution mixed with Gadovist contrast (1:100) was administered *via* the intra-arterial catheter under dynamic magnetic resonance imaging to assess the transcatheter brain perfusion territory. All animals underwent routine brain MRI, including anatomical T2, DTI, and PWI scans. Stroke evolution was monitored using MRI for up to 2 h after thrombin injection. After 2 h, tPA (average 0.58 mg/kg, total dose 20 mg at a concentration 1 mg/ml) was administered using the same intra-arterial catether, and another PWI scan was performed to verify reperfusion. PWI was carried out using a timed contrast bolus passage technique. The contrast agent (Gadovist 1.0) was injected into an auricular vein via a 20-gauge intravenous cannula at a flow rate of 3 mL/s ~7 s after the acquisition began. Other parameters for the PWI sequences were as follows: repetition time/echo time = 1,850/45 ms, acquisition matrix = 128 × 128, slice thickness = 5 mm. Two animals were excluded due to technical issues with contrast injection and suboptimal perfusion scans.

Follow-up MRI was done 7 and 28 days post stroke induction.

Behavioral testing (Neurological Evaluation Grading Scale) was performed on day 3, 7, and 28 after stroke to assess functional outcome after ischemia (Tanaka et al., 2008).

Animals were sacrificed 28 days after stroke induction (under anesthesia, immediately after MR imaging) by intravenous administration of sodium pentobarbital at a dose 140 mg/kg.

### Image analysis

Image analysis was done by two independent blinded researchers. PWI: Perfusion maps (CBV, CBF, TTP, Tmax) and volumetric analysis were performed with IB Neuro^TM^ and IB DCE^TM^, and IB DeltaSuite^TM^ toolkits (Imaging Biometrics, USA) within the Horos platform. Circular ROIs were outlined for corresponding regions in the ipsilateral and contralateral hemispheres, and the relative signal was measured using Osirix Lite. Apparent Diffusion Coefficient (ADC) maps were generated with AW Volume Share software, and image analysis was made using the ImageJ software package (National Institutes of Health).

### Histological analysis

Twenty-eight days after stroke induction brain tissue was perfused with 4% paraformaldehyde followed by 4 % saccharose, cryopreserved for 1 week with sucrose and then stored in −80 degrees Celsius for further analysis. Brain samples were sectioned using cryostat into 30-micrometer thick slices and stained with hematoxylin and eosin for general tissue morphology assessment. Additionally, immunostaining was performed for specific cellular markers, including NeuN (abcam, cat. ab177487) for neurons, GFAP (Invitrogen, cat. 13-0300) for astrocytes, IBA-1 (Wako/Fuji Film, cat. 019-19741)for microglia. DAPI was utilized as a nuclear counterstain. To achieve this, the tissue sections were incubated over night at 60°C in 10 mM citrate buffer with 0.05% Tween-20 at pH 6. Following this antigen-retrieval step, the sections were washed and blocked in TBS (50 mM Tris Base, 150 mM NaCl, pH 7.6) with 10% normal donkey serum and 1% bovine serum albumin for 1 h at room temperature. The sections were then incubated with the following primary antibodies over night at 4°C: rabbit anti-NeuN (Abcam, UK), rat anti-GFAP (Merck, Germany), and rabbit anti-IBA-1 (FUJIFILM Wako Pure Chemical Corporation, USA). After washing with TBS, the sections where then labeled with donkey secondary antibodies coupled to Alexa Fluor 488 or 555 directed against rabbit or rat IgG (Thermo Fisher Scientific, USA) for 2 h at room temperature. To stain cell nuclei, 1μg/ml DAPI was added to the secondary antibody solution. After a final wash, the sections were mounted in Shandon™ Immu-Mount™ (Thermo Fisher Scientific, USA) and imaged on a Leica SPE fluorescence microscope (Leica, Germany).

### Statistical analysis

All data were analyzed using GraphPad Prism 8 Software (GraphPad, San Diego, USA) and presented as mean ± SD. Perfusion parameters after thrombin and tPA were normalized to the contralateral hemisphere at the same time points. The *in vitro* data were analyzed using two-way ANOVA followed by multiple comparisons, and the results of the *in vivo* experiment were calculated using an paired Student's *t*-test. Differences were considered statistically significant at the 95% confidence level (*p* < 0.05).

## Results

### *In vitro* tPA thrombolytic activity with a porcine blood clot

**Figure 2:**
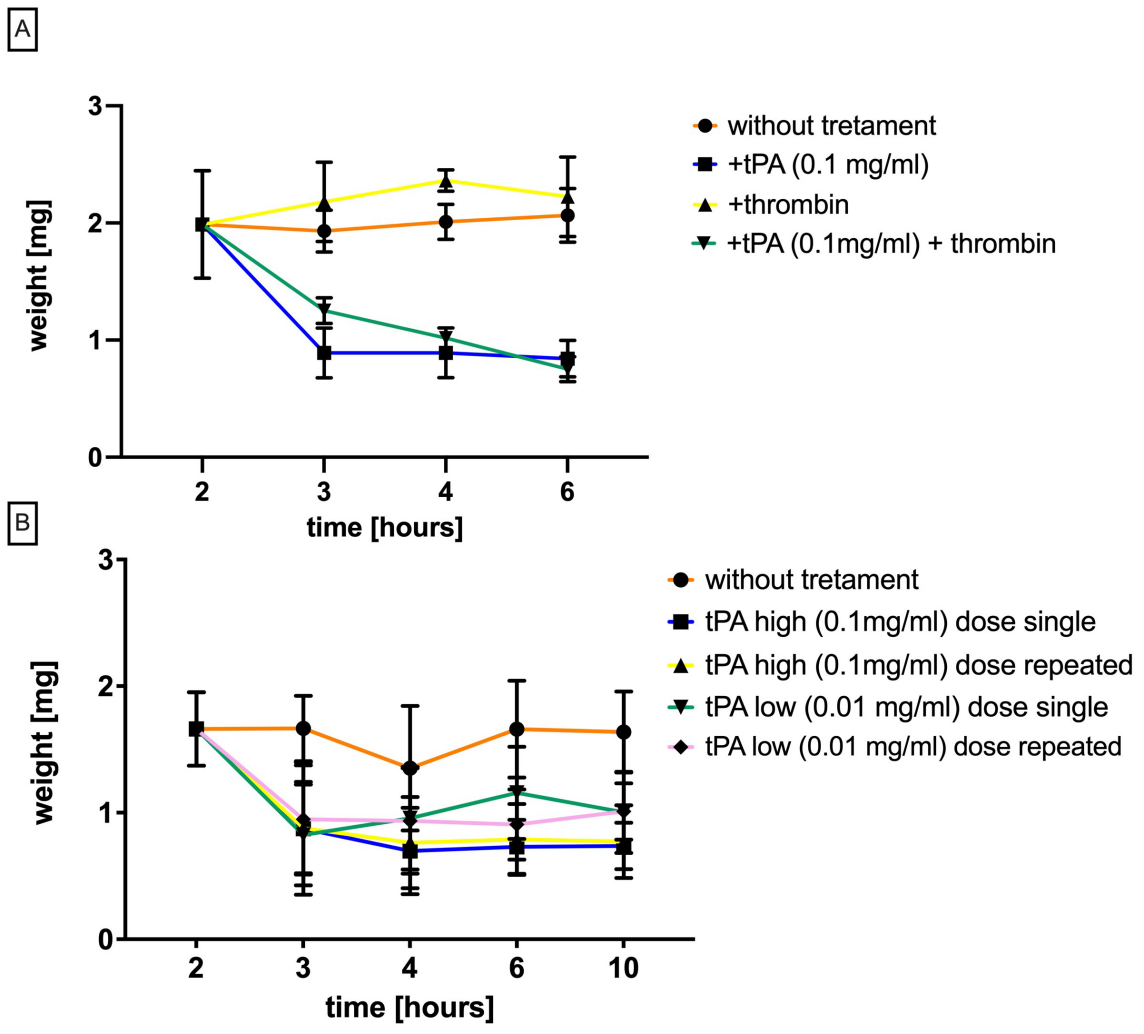
*In vitro* evaluation of tPa on porcine clots. **(A)** thrombin induced clot changes after tPA treatment **(B)** clot weight changes after tPA treatment. Differences were considered statistically significant at the 95% confidence level (*p* < 0.05).

This study aimed to investigate the efficacy of tPA, a commonly used thrombolytic agent in stroke treatment, in dissolving clots in pig blood (*n* = 5). Two separate assays were conducted: one with natively coagulated blood and the other with blood pre-treated with thrombin. The results of the native clot assay demonstrated that tPA had a potent thrombolytic effect on porcine clots, with statistically significant reductions (*p* < 0.0001) in clot weight compared to untreated samples. No significant differences were observed in response to varying doses or re-dosings of tPA. Notably, the greatest reduction in clot weight occurred between the second and third hour following the first tPA administration (Figure 2B). The second *in vitro* experiment evaluated the effect of adding thrombin to the blood samples during the thrombolysis process, but no significant differences were observed between tPA alone vs. thrombin plus tPA (Figure 2A).

### Induction of stroke and reperfusion

**Figure 3:**
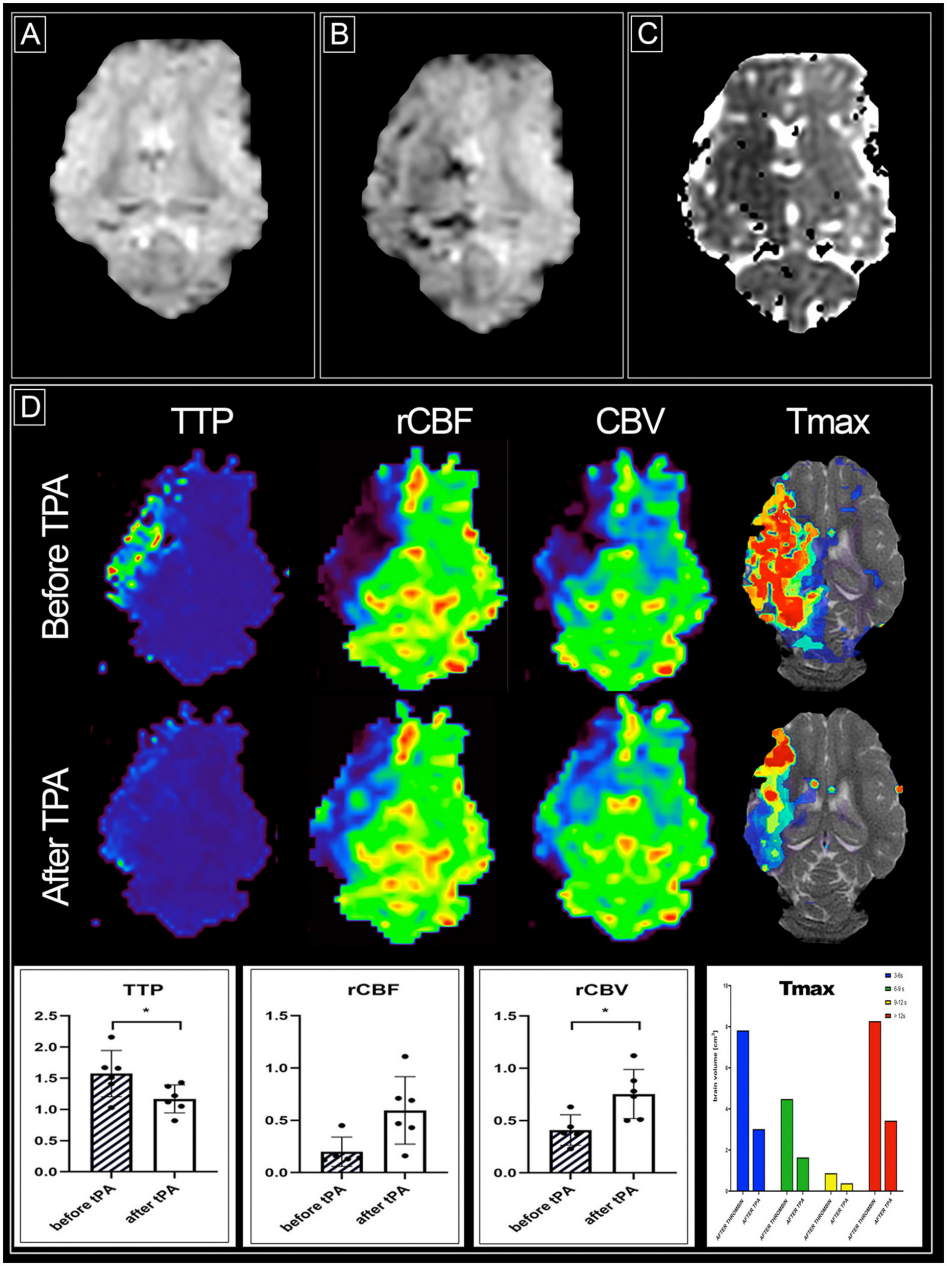
Stroke induction and reperfusion evaluation using magnetic resonance imaging. **(A)** baseline susceptibility weighted image (SWI); **(B)** SWI after thrombin injection showing retention of the contrast indicative of cerebral vasculature occlusion; **(C)** ADC map after stroke induction with hypointense region corresponding to stroke lesion; **(D)** parametric MRI perfusion maps before and after tPA injection with quatitative evaluation. Differences were considered statistically significant at the 95% confidence level (p <0.05). Statistical analysis: ^*^p <0.05.

The success rate of cerebral ischemia induction with thrombin was 100%. Blockage of cerebral vasculature was verified under real-time MRI as evidenced by hypointense regions on gradient echo scans (Figure 3B) in comparison to baseline scan (Figure 3A). With the induction of stroke inside the MRI scanner, we were able to monitor brain injury with diffusion-weighted scans, and ADC maps detected abnormalities as early as 10 min after thrombin injection (Figure 3C). Hypointense regions in the ipsilateral hemisphere and measured signal intensity for ROIs on ADC maps within the lesion vs. the contralateral hemisphere measured as mean ± SD were at 85.520 ± 12.779 vs. 99.863 ± 3.138, respectively. The average volume of brain lesions on ADC maps was 8.412±6.799 cm^3^, with the total brain volume of a pig ~100 cm^3^.

Perfusion MRI and the generated parametric maps showed hypoperfusion in the ipsilateral hemisphere, primarily in the watershed of the middle cerebral artery (Figure 3D). Pharmacological intra-arterial thrombolysis resulted in the reversal of vasculature blockage. There was a normalization of perfusion parameters with TTP and T max volume at the delay of > 6 s, providing the most striking difference. For quantitative ROI assessment, intensity on a parametric map was measured (mean ± SD) for TTP before and after tPA and was 1.573 ± 0.371 and 1.168 ± 0.223, respectively. Cerebral blood volume decreased significantly after thrombin administration, and thrombolysis significantly restored perfusion within the ischemic region (0.517 ± 0.292 and 0.753 ± 0.235, respecrtively). Cerebral blood flow before tPA was at 0.278 ± 0.229, and following reperfusion, it reached 0.595 ± 0.322 with an almost three-fold increase. During this acute phase, up to 2 h after induction of ischemia, no abnormalities on T2 weighted or FLAIR scans were observed. Table 1 presents perfusion outcomes per animal.

**Table 1:** Summary table for perfusion outcomes per animal.

	**ADC**		**TTP**		**CBF**		**CBV**	
**Animal no**.	**After thrombin**	**After tPA**	**After thrombin**	**After tPA**	**After thrombin**	**After tPA**	**After thrombin**	**After tPA**
#1	66.907	96.138	1.67	1.12	0.13	0.69	0.44	0.73
#2	80.435	101.481	1.41	1.37	0.45	0.47	1.05	1.12
#3	93.285	99.178	2.16	1.43	0.12	0.16	0.38	0.51
#4	104.949	104.803	1.03	1.05	0.67	0.71	0.63	0.78
#5	84.168	100.457	1.51	0.82	0.16	1.11	0.37	0.88
#6	83.375	97.121	1.66	1.22	0.14	0.43	0.23	0.5
Mean	85.520	99.863	1.573	1.168	0.278	0.595	0.517	0.753
SD	12.779	3.138	0.371	0.223	0.229	0.322	0.292	0.235

### Long-term outcomes for individual animals following induction of stroke with reperfusion

**Figure 4:**
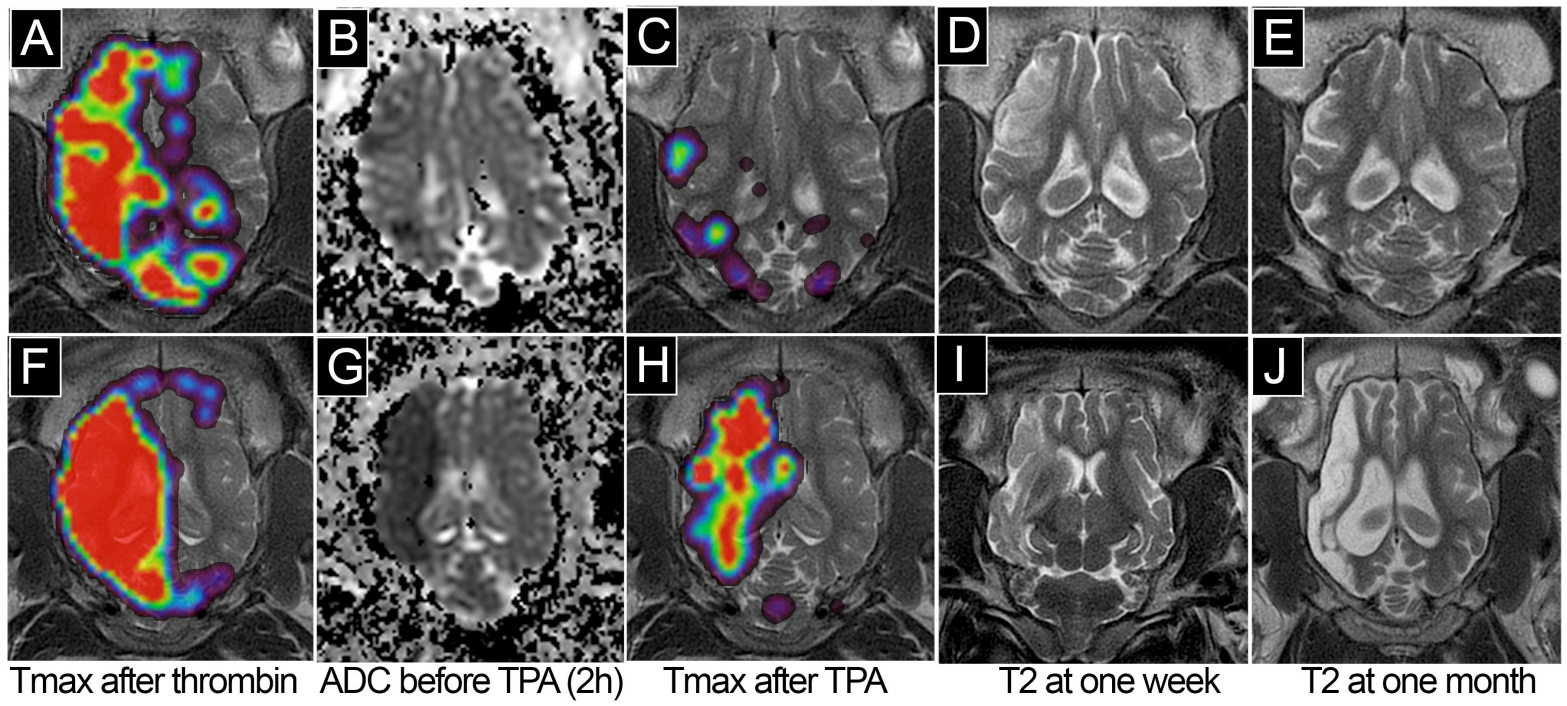
Longitudinal assessment of stroke evolution in pigs with mild **(A–E)** vs. severe **(F–J)** stroke. Parametric MRI perfusion T-max map before **(A, F)** and after **(C, H)** tPA; ADC map **(B, G)**; T2 7 days **(D, I)**, and 1 month **(E, J)** after stroke.

Injection of thrombin under real-time MRI guidance provides excellent control over the severity to cerebral vasculature occlusion, allowing for tuning of the size of stroke lesions. In a representative pig with moderate occlusion, as measured by parametric Tmax maps, the brain volume with perfusion at Tmax >6 s after thrombin injection was 11.52 cm^3^ (Figure 4A). This corresponded to ADC hypointensity at 6.800 cm^3^ (Figure 4B). Tmax >6 s after tPA injection dropped to 1.55 cm^3^ after IA TPA (Figure 4C) indicating an excellent reperfusion effect. Follow-up MRI at 1 week showed T2 hyperintensity in a volume of 5.320 cm^3^ (Figure 4D), which decreased to 1.270 cm^3^ at 1 month (Figure 4E). In another pig with severe occlusion, the brain volume with Tmax >6 s after thrombin was 16.95 cm^3^ (Figure 4F), which corresponded to ADC hypointensity at 21.610cm^3^ (Figure 4G). After TPA again we observed reduction of Tmax >6 s (Figure 4H). At 1 week, the region of T2 hyperintensity was 16.890 cm^3^ (Figure 4I); at 1 month, it was 13.830cm^3^ with noticeable brain atrophy (Figure 4J). Table 2 summarize lesion volumes per animal.

**Table 2:** Summary table with lesion volumes per animal.

	**ADC**	**T2**	**T2**
**Animal no**.	**Lesion size**	**Day 7**	**Day 28**
#1	21.61 cm^3^	16.89 cm^3^	13.83 cm^3^
#2	6.8 cm^3^	5.32 cm^3^	1.27 cm^3^
#3	8.46 cm^3^	9.62 cm^3^	5.35 cm^3^
#4	6.54 cm^3^	7.54 cm^3^	5.53 cm^3^
#5	2.27 cm^3^	1.51 cm^3^	1.09 cm^3^
#6	4.79 cm^3^	17.58 cm^3^	11.45 cm^3^
Mean	8.412	9.743	6.420
SD	6.799	6.399	5.236

All surviving animals were ambulatory and capable of eating and drinking independently; however, they exhibited significant neurological motor deficits in the contralateral limbs. Following stroke induction, the animals showed rapid fatigue and apathy, frequently tilted their heads to one side, experienced difficulty walking, appeared disoriented, and often lost balance and coordination. Other than neurological deficits anticipated after stroke we did not observe any adverse events. Table 3 presents neurological scores per animal in current study.

**Table 3:** Neurologival score per animal.

	**Neurological score (maximum 23 points)**		
**Animal no**.	**Day 3**	**Day 7**	**Day 28**
#1	12	4	0
#2	1	1	0
#3	2	0	0
#4	2	0	0
#5	0	0	0
#6	2	0	1

**Figure 5:**
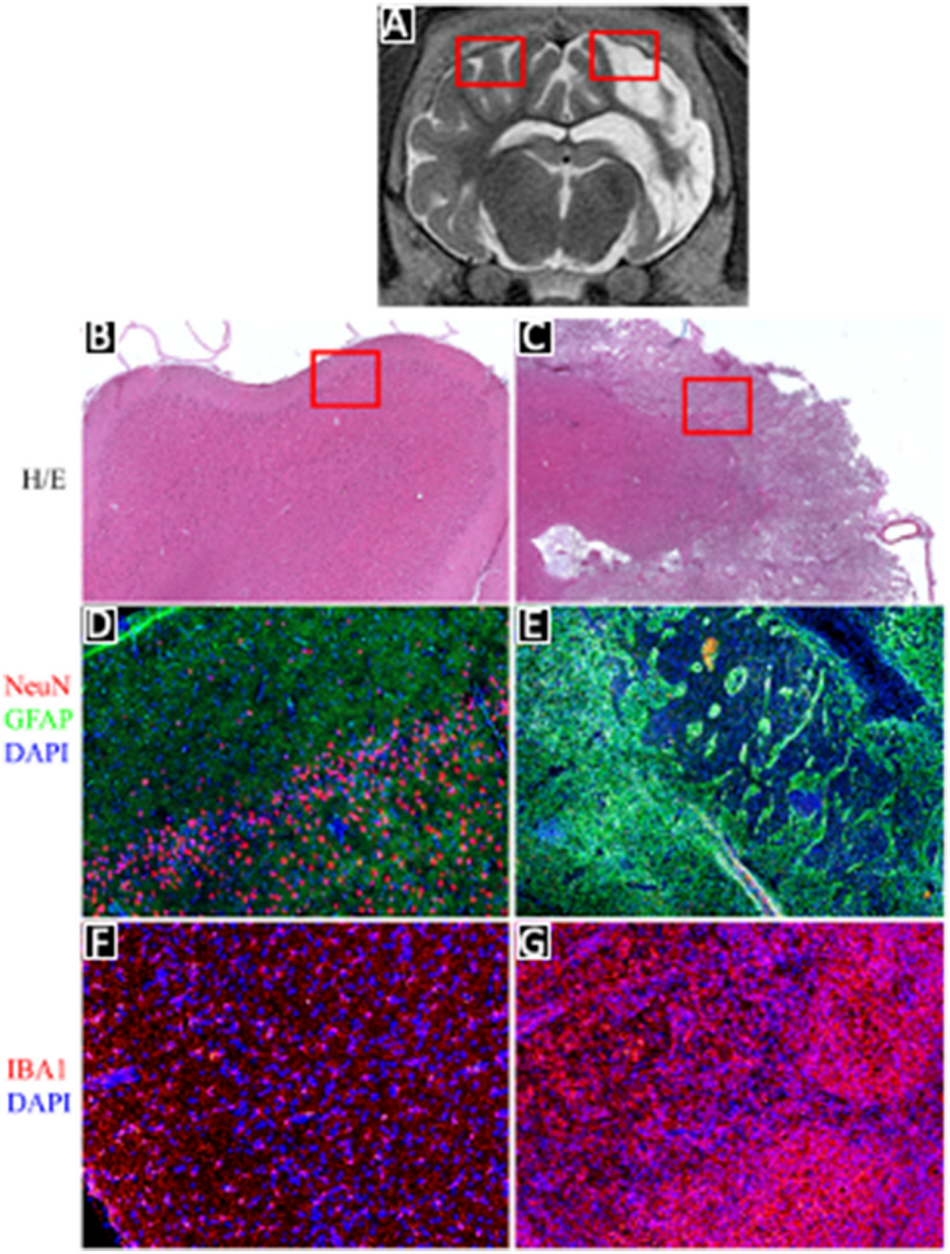
Histologial evaluation of pig brain after stroke. **(A)** MRI coronal T2 section of pig brain after stroke followed by reperfusion; **(B, C)** H&E staining of contralateral and ipsilateral hemisphere; **(D, E)** NeuN and GFAP immunofluorescence staining of contralateral and ipsilateral hemisphere; **(F, G)** Iba1 immunofluorescence staining of contralateral and ipsilateral hemisphere.

Histological analysis conducted 1 month after stroke induction revealed distinct findings in the region corresponding to T2 hyperintensity observed in MRI (Figure 5A). Hematoxylin and eosin staining depicted a characteristic pattern consistent with complete disruption of brain ultrastructure and the formation of a scar (Figure 5C), contrasting with the normal brain architecture observed in the corresponding area of the contralateral hemisphere (Figure 5B). Immunohistochemistry targeting neurons (NeuN, red) and astrocytes (GFAP, green) demonstrated cortical neurons and quiescent astroglia in the contralateral hemisphere (Figure 5D), whereas the lesion site exhibited complete neuronal loss, replaced by an astrocytic scar (Figure 5E). Additionally, staining for the microglial marker IBA-1 revealed typical ramified resting microglia in the contralateral hemisphere (Figure 5F), whereas the lesion site showed activation, hypertrophy, and an increased number of microglia (Figure 5G).

## Discussion

Focal ischemic stroke by middle cerebral artery occlusion in large animal models can be performed endovascularly using a catheter-based approach or surgically with an open craniotomy approach. Both methods can be used for permanent and transient occlusion. However, until recently, endovascular models with reperfusion were only possible in dogs and NHPs, while anatomical constrains in swine and sheep prevented vascular access to brain arteries (Taha et al., 2022). Specifically, pigs possess a unique vascular structure called the rete mirabile, a dense network of small vessels located at the skull base that impedes direct catheter-based navigation to the intracranial arteries, thereby preventing the use of traditional endovascular techniques commonly employed in clinical stroke interventions. Here, we presented an effective strategy to circumvent this limitation, expanding the utility of pigs as an ischemic stroke model. This is particularly advantageous as pigs offer several critical advantages. Their large gyrencephalic brain closely resembles the human brain in terms of structure, size, and cortical complexity, facilitating translation of imaging protocols, surgical techniques, and intervention strategies. Moreover, pigs display physiological and metabolic profiles similar to humans, which can enhance the relevance of stroke pathophysiology, inflammation, immune response, and blood-brain barrier dynamics observed in these models (Fisher et al., 2009).

In our previous study (Golubczyk et al., 2020), we introduced a novel model of focal ischemic stroke in swine induced by intraarterial injection of thrombin, which was monitored by real-time MRI. In this updated work, we extend our approach by demonstrating for the first time in pigs the feasibility of pharmacological recanalization via intra-arterial rtPA delivery and confirm its efficacy using continuous MRI-based perfusion imaging. Unlike many large animal stroke models, our approach facilitates high spatiotemporal resolution monitoring of stroke evolution in real time, setting a new benchmark for preclinical stroke research. One unique aspect of our approach is using x-ray angiography for catheter navigation and MRI for monitoring stroke induction and evolution in real time, providing a comprehensive view of the stroke process. Compared to our last work, we replaced an expensive microcatheter with a low-cost 4F catheter, facilitating widespread model dissemination.

Several previously reported large animal models of stroke have demonstrated important progress in this area, but especially those in swine do not show data on recanalizing vessels and lack the ability to dynamically track cerebral perfusion. For example, Røhl et al. (2002) used MRI to image stroke lesions in pigs but employed surgical clip occlusion without reperfusion and terminated the animals after imaging at 7 h, limiting clinical relevance due to absence of thromboembolic mechanisms. Similarly, Sakoh et al. (2001) applied PET and MRI to assess tissue viability but used a permanent MCA occlusion model. In contrast, our model employs a thrombin-induced occlusion to better recapitulate human thromboembolic stroke and allows pharmacologic reperfusion with intra-arterial rtPA, closely mimicking clinical workflows. One of the few studies offering a comparable level of real-time monitoring and reperfusion control is the work by Wu et al. (2022), who established an embolic stroke model in rhesus monkeys using autologous clots delivered via microcatheter. Like our approach, their model allows intra-arterial thrombolysis with rtPA. However, the use of non-human primates presents ethical and practical constraints, including high costs and limited availability. In another related study, Wright et al. (2016) used CT perfusion and F-18-FFMZ-PET imaging in a porcine model of ischemia to define infarct thresholds. However, they relied on ET-1-induced occlusion, which does not allow for thrombolysis or recanalization, and perfusion imaging was not used to guide or assess therapy in real time. Dogs have also been used for endovascular induction of stroke (Atchaneeyasakul et al., 2024) and although easier to manage compared to primates, have cerebral vasculature characterized by significant tortuosity and kinking, which complicates catheter navigation (Lv et al., 2020). Thus, while each animal model brings its own set of advantages and limitations, pigs remain particularly attractive for translational stroke research as the existing anatomical limitations of the rete mirabile are being effectively addressed. The novelty of our model lies not only in the feasibility of endovascular stroke induction and reperfusion in swine, but also in the integration of high-resolution perfusion imaging at multiple time points. We utilized x-ray angiography to navigate the endovascular catheter to the brain-supplying pharyngeal ascending artery and real-time MRI to monitor both ischemia and reperfusion (Walczak et al., 2017). Through imaging, we were able to fully document the feasibility and reproducibility of intra-arterial alteplase-induced reperfusion. This enables a highly controlled evaluation of ischemic injury progression, therapeutic response, and long-term tissue outcomes. The ability to document partial or complete reperfusion with quantitative metrics (e.g., Tmax, rCBF, rCBV) mirrors clinical assessments used in stroke trials and provides direct translational relevance. Moreover, our MRI-based verification of perfusion deficits and recanalization aligns with the standard criteria used in human studies such as the DEFUSE 2 trial (Marks et al., 2014), where Tmax maps were critical for assessing penumbra and predicting tissue outcomes. The capability to recapitulate these metrics in a large animal model positions our system for use in evaluating experimental thrombolytics, adjuvant therapies, and neuroprotectants.

While embolic models in rodents have generated vast preclinical datasets, their limited anatomical and physiological similarity to humans has hampered translational progress (Savitz et al., 2011). This issue of ineffective translational stroke research has been addressed by the Stroke Therapy Academic Industry Roundtable, which recommends using at least two species for translational stroke therapies (Fisher et al., 2009). For initial testing, rodents or rabbits are acceptable. However, large gyrencephalic animals such as dogs, sheep, pigs, or non-human primates (NHPs) are desirable for more advanced efficacy studies. Our swine model fulfills this role and adds the capacity for real-time monitoring and intervention, features not previously demonstrated in comparable porcine stroke studies.

Swine are commonly used to study fibrinolytic treatment of pulmonary embolism, although evidence suggests that pig clots may be resistant to alteplase-induced thrombolysis (Flight et al., 2006). To investigate the utility of alteplase for our reperfusion study, we conducted a series of *in vitro* tests and observed a 50% reduction in clot weight after 2 h of tPA treatment. We also found that the lysis of thrombin-enriched clots was not affected. Interestingly, our results differ from those of Evandro et al. (2021), who reported a weak thrombolytic effect against swine blood clots compared to human blood clots, with only higher concentrations of alteplase resulting in nearly 80% lysis in both human and swine clots. Our study found that neither the dose of alteplase nor repeated dosing had any additional effect. The consistency observed across dosing strategies likely reflects inherent characteristics of our *in vitro* assay, which lacks the dynamic conditions present *in vivo*, such as active blood flow, thrombus mechanical forces, and clearance mechanisms, all of which strongly influence clot dissolution kinetics. Thus, the absence of complete dissolution can be reasonably attributed to the intrinsic limitations of our simplified setup. Furthermore, this observation aligns well with prior reports in the literature indicating that *in vitro* clot lysis assays typically reach a plateau in thrombus dissolution due to accumulation of fibrinolysis-inhibiting by-products, spatial constraints limiting enzyme diffusion, or saturation of plasminogen binding sites within the thrombus structure (Ammollo et al., 2010). Such phenomena would inherently limit maximal achievable clot mass reduction.

Our study addresses a critical gap in translational stroke research by establishing a reproducible embolic stroke model in swine that uniquely integrates real-time MRI-guided monitoring with clinically relevant pharmacologic recanalization. Compared to previously reported large animal models, many of which rely on craniotomy, electrocoagulation, or embolic methods without reperfusion capability, our approach offers three key advantages: (1) non-invasive endovascular induction of thrombosis that closely mimics human stroke etiology; (2) continuous, high-resolution imaging throughout the ischemic and reperfusion phases; and (3) the ability to evaluate therapeutic interventions in a controlled and clinically reflective setting.

This platform provides a valuable tool for studying acute ischemic stroke and for preclinical validation of novel interventions such as neuroprotectants, stem cells, gene therapies or exosomes (Waseem et al., 2023). Swine, with their large, gyrencephalic brains and human-like white-to-gray matter ratios, offer superior anatomical and physiological fidelity over rodents or rabbits. Furthermore, leveraging multimodal imaging technologies such as x-ray angiography and advanced MRI ensures highly reproducible stroke induction and precise quantification of lesion evolution and therapeutic effects.

It is worth noting that human stroke is most frequently caused by cerebral thromboembolism. Therefore, the thromboembolic model is most appropriate for studying reperfusion among animal ischemic stroke models, as it closely resembles the clinical situation. In human ischemic stroke, spontaneous or intervention-induced reperfusion occurs gradually over time, and this process is best represented in the embolic stroke model. In contrast, external clip occlusion or intraluminal filament occlusion models represent sudden reperfusion events. Furthermore, our thromboembolic swine model closely mimics human pathophysiology, including cytotoxic and vasogenic edema, BBB impairment, and the presence of penumbra; this last feature is particularly prominent in cases with less severe initial occlusion. Cerebral ischemia-reperfusion injury is a complex cascade of pathophysiological events that can lead to irreversible neuronal injury, including energy failure, elevated intracellular calcium, excitotoxicity, free radical generation, BBB disruption, edema, and apoptosis (Li et al., 2022). While recanalization, whether spontaneous or intervention-induced, can halt the devastating effects of hypoxia by restoring oxygenated blood flow, it is also known to initiate the cascade of secondary brain injury. Therefore, it is crucial to study stroke and therapeutic interventions in a setting that accurately models both ischemia and reperfusion to develop effective treatments for stroke patients.

## Limitations

While our study presents a significant advancement in modeling both ischemia and reperfusion in a clinically relevant porcine model of stroke, several limitations must be acknowledged. First, although the recanalization achieved with intra-arterial rtPA administration led to significant improvements in perfusion parameters and demonstrated vessel reopening, it did not always result in full restoration of cerebral perfusion to baseline or contralateral levels. This partial reperfusion is an anticipated and clinically relevant outcome, as even complete clot dissolution does not necessarily guarantee physiological perfusion recovery. Secondly, while the inclusion of untreated controls would have provided comparative insights into spontaneous stroke evolution, our study design utilized each animal as its own internal control. High-resolution MRI performed throughout the procedure enabled precise tracking of perfusion changes before and after thrombolysis, including intrasubject comparison to the contralateral hemisphere and baseline pre-stroke scans. Another important consideration is the use of young, healthy pigs rather than aged or comorbid animals, which may not fully reflect the clinical heterogeneity seen in human stroke populations. Finally, due to the significant influence of systemic physiological parameters on stroke outcomes, we introduced pharmacological blood pressure reduction during the stroke induction phase to lower baseline perfusion pressure. While this allowed us to create consistent ischemic lesions, it introduces a variable that may not fully replicate spontaneous stroke conditions in humans. Notably, transient hypotension might induce early-phase ischemic preconditioning, which could provide short-term neuroprotective effects and may influence lesion development and treatment response (Qiu et al., 1997). Future studies will investigate more physiologically adaptive perfusion modulation strategies to refine model fidelity.

Finally, the intra-arterial route of drug administration modeled here aligns with emerging clinical strategies for targeted cerebral delivery, allowing for rapid and localized exposure of ischemic tissue to high drug concentrations. We have previously demonstrated this approach's feasibility in our model through successful intra-arterial delivery of glycolic acid during the acute stroke phase (Chovsepian et al., 2022), and this study further extends that capability to thrombolytic therapy. Collectively, our model offers a translationally robust, imaging-rich, and therapeutically adaptable system that can serve as a critical intermediary step between rodent studies and human clinical trials.

## Data Availability

The raw data supporting the conclusions of this article will be made available by the authors, without undue reservation.

## References

[B1] Ammollo C. T. Semeraro F. Incampo F. Semeraro N. Colucci M. (2010). Dabigatran enhances clot susceptibility to fibrinolysis by mechanisms dependent on and independent of thrombin-activatable fibrinolysis inhibitor. J. Thromb. Haemost. 8, 790–798. 10.1111/j.1538-7836.2010.03739.x 20088944

[B2] Armstead W. Riley J. Yarovoi S. Higazi A. A. Cines D. B. (2016). tPA Variant tPA-A296-299 prevents impairment of cerebral autoregulation after stroke through lrp dependent increase in cAMP and p38 MAPK. Stroke 47. 10.1161/STROKEAHA.116.012678 27354223 PMC4961526

[B3] Armstead W. M. Ganguly K. Riley J. Zaitsev S. Cines D. B. Higazi A. A.. (2012). RBC-coupled tPA prevents whereas tPA aggravates JNK MAPK-mediated impairment of ATP- and Ca-sensitive K channel-mediated cerebrovasodilation after cerebral photothrombosis. Transl. Stroke Res. 3, 114–121. 10.1007/s12975-011-0105-1 23577046 PMC3619434

[B4] Armstead W. M. Riley J. Kiessling J. W. Cines D. B. Higazi A. A. (2010). Novel plasminogen activator inhibitor-1-derived peptide protects against impairment of cerebrovasodilation after photothrombosis through inhibition of JNK MAPK. Am. Journal of Physiol.-Regul. Integrat. Comparat. Physiol. 299, R480–R485. 10.1152/ajpregu.00256.2010 20538898 PMC2928614

[B5] Atchaneeyasakul K. Valasaki K. Silvera R. Khan A. Yavagal D. (2024). Optimal technique for canine mesenchymal stem cells labeling with novel SPIO, MIRB: for MRI detection of transplanted stem cells canine stroke model. Neurol. Res. 46, 326–329. 10.1080/01616412.2024.2303879 38468486

[B6] Chovsepian A. Berchtold D. Winek K. Mamrak U. Ramírez Álvarez I. Dening Y.. (2022). A primeval mechanism of tolerance to desiccation based on glycolic acid saves neurons in mammals from ischemia by reducing intracellular calcium-mediated excitotoxicity. Adv Sci (Weinh) 9, e2103265. 10.1002/advs.202103265 34904402 PMC8811841

[B7] d'Esterre C. D. Aviv R. I. Morrison L. Fainardi E. Lee T. Y. (2015). Acute multi-modal neuroimaging in a porcine model of endothelin-1-induced cerebral ischemia: defining the acute infarct core. Transl. Stroke Res. 6, 234–241. 10.1007/s12975-015-0394-x 25876960

[B8] Elliott J. T. Diop M. Morrison L. B. d'Esterre C. D. Lee T. Y. St Lawrence K. (2014). Quantifying cerebral blood flow in an adult pig ischemia model by a depth-resolved dynamic contrast-enhanced optical method. Neuroimage 94, 303–311. 10.1016/j.neuroimage.2014.03.023 24650601

[B9] Evandro M. Neto-Neves D. M. B. Kline J. A. (2021). The resistance of swine blood clots to alteplase-induced thrombolysis *in vitro* is concentration-dependent. Thrombosis Update 2:100035. 10.1016/j.tru.2021.100035

[B10] Fisher M. Feuerstein G. Howells D. W. Hurn P. D. Kent T. A. Savitz S. I.. (2009). Update of the stroke therapy academic industry roundtable preclinical recommendations. Stroke 40, 2244–2250. 10.1161/STROKEAHA.108.541128 19246690 PMC2888275

[B11] Flight S. M. Masci P. P. Lavin M. F. Gaffney P. J. (2006). Resistance of porcine blood clots to lysis relates to poor activation of porcine plasminogen by tissue plasminogen activator. Blood Coagul. Fibrinolysis 17, 417–420. 10.1097/01.mbc.0000233374.79593.57 16788320

[B12] Frenkel V. Oberoi J. Stone M. J. Park M. Deng C. Wood B. J.. (2006). Pulsed high-intensity focused ultrasound enhances thrombolysis in an *in vitro* model. Radiology 239, 86–93. 10.1148/radiol.2391042181 16493016 PMC2386885

[B13] GBD 2019 Stroke Collaborators (2021). Global, regional, and national burden of stroke and its risk factors, 1990-2019: a systematic analysis for the Global Burden of Disease Study 2019. Lancet Neurol 20, 795–820. 10.1016/S1474-4422(21)00252-0 34487721 PMC8443449

[B14] Golubczyk D. Kalkowski L. Kwiatkowska J. Zawadzki M. Holak P. Glodek J.. (2020). Endovascular model of ischemic stroke in swine guided by real-time MRI. Sci. Rep. 10, 17318. 10.1038/s41598-020-74411-3 33057149 PMC7560864

[B15] Knight R. A. (2000). Cerebral blood flow and blood volume measured by magnetic resonance imaging bolus tracking after acute stroke in pigs - Comparison with [O-15]H2O positron emission tomography - Editorial comment. Stroke 31, 1964–1964. 10.1161/01.STR.31.8.1958 10926964

[B16] Kuluz J. W. Prado R. He D. Zhao W. Dietrich W. D. Watson B. (2007). New pediatric model of ischemic stroke in infant piglets by photothrombosis: acute changes in cerebral blood flow, microvasculature, and early histopathology. Stroke 38, 1932–1937. 10.1161/STROKEAHA.106.475244 17463315

[B17] Lesniak W. G. Chu C. Jablonska A. Behnam Azad B. Zwaenepoel O. Zawadzki M.. (2019a). PET imaging of distinct brain uptake of a nanobody and similarly-sized PAMAM dendrimers after intra-arterial administration. Eur. J. Nucl. Med. Mol. Imaging 46, 1940–1951. 10.1007/s00259-019-04347-y 31161257 PMC7571511

[B18] Lesniak W. G. Chu C. Jablonska A. Du Y. Pomper M. G. Walczak P.. (2019b). A distinct advantage to intraarterial delivery of (89)Zr-bevacizumab in PET imaging of mice with and without osmotic opening of the blood-brain barrier. J. Nucl. Med. 60, 617–622. 10.2967/jnumed.118.218792 30315146 PMC6495238

[B19] Li G. Ye C. Zhu Y. Zhang T. Gu J. Pan J.. (2022). Oxidative injury in ischemic stroke: a focus on NADPH oxidase 4. Oxid. Med. Cell. Longev. 2022, 1148874. 10.1155/2022/1148874 35154560 PMC8831073

[B20] Lv X. Li C. Jiang W. (2020). The intracranial vasculature of canines represents a model for neurovascular ischemia and training residents and fellows in endovascular neurosurgery. Neuroradiol. J. 33, 292–296. 10.1177/1971400920920787 32367763 PMC7416350

[B21] Mangla S. Choi J. H. Barone F. C. Novotney C. Libien J. Lin E.. (2015). Endovascular external carotid artery occlusion for brain selective targeting: a cerebrovascular swine model. BMC Res. Notes 8, 808. 10.1186/s13104-015-1714-7 26689288 PMC4687072

[B22] Marks M. P. Lansberg M. G. Mlynash M. Kemp S. McTaggart R. A. Zaharchuk G.. (2014). Angiographic outcome of endovascular stroke therapy correlated with MR findings, infarct growth, and clinical outcome in the DEFUSE 2 trial. Int J Stroke 9, 860–865. 10.1111/ijs.12271 24684804 PMC4411961

[B23] Qiu Y. Tang X. L. Park S. W. Sun J. Z. Kalya A. Bolli R. (1997). The early and late phases of ischemic preconditioning: a comparative analysis of their effects on infarct size, myocardial stunning, and arrhythmias in conscious pigs undergoing a 40-minute coronary occlusion. Circ. Res. 80, 730–742. 10.1161/01.RES.80.5.730 9130454

[B24] Renú A. Millán M. San Román L. Blasco J. Martí-Fàbregas J. Terceño M.. (2022). Effect of intra-arterial alteplase vs placebo following successful thrombectomy on functional outcomes in patients with large vessel occlusion acute ischemic stroke: the CHOICE randomized clinical trial. JAMA 327, 826–835. 10.1001/jama.2022.1645 35143603 PMC8832304

[B25] Rha J. H. Saver J. L. (2007). The impact of recanalization on ischemic stroke outcome: a meta-analysis. Stroke 38, 967–973. 10.1161/01.STR.0000258112.14918.24 17272772

[B26] Røhl L. Sakoh M. Simonsen C. Z. Vestergaard-Poulsen P. Sangill R. Sørensen J. C.. (2002). Time evolution of cerebral perfusion and apparent diffusion coefficient measured by magnetic resonance imaging in a porcine stroke model. J. Magnet. Resonance Imag. 15, 123–129. 10.1002/jmri.10068 11836766

[B27] Sakoh M. Ostergaard L. Gjedde A. Røhl L. Vestergaard-Poulsen P. Smith D. F.. (2001). Prediction of tissue survival after middle cerebral artery occlusion based on changes in the apparent diffusion of water. J. Neurosurg. 95, 450–458. 10.3171/jns.2001.95.3.0450 11565867

[B28] Sakoh M. Ostergaard L. Røhl L. Smith D. F. Simonsen C. Z. Sørensen J. C.. (2000). Relationship between residual cerebral blood flow and oxygen metabolism as predictive of ischemic tissue viability: sequential multitracer positron emission tomography scanning of middle cerebral artery occlusion during the critical first 6 hours after stroke in pigs. J. Neurosurg. 93, 647–657. 10.3171/jns.2000.93.4.0647 11014544

[B29] Savitz S. I. Chopp M. Deans R. Carmichael T. Phinney D. Wechsler L.. (2011). Stem cell therapy as an emerging paradigm for stroke (STEPS) II. Stroke 42, 825–829. 10.1161/STROKEAHA.110.601914 21273569

[B30] Schöll M. J. Santos E. Sanchez-Porras R. Kentar M. Gramer M. Silos H.. (2017). Large field-of-view movement-compensated intrinsic optical signal imaging for the characterization of the haemodynamic response to spreading depolarizations in large gyrencephalic brains. J. Cereb. Blood Flow Metabol. 37, 1706–1719. 10.1177/0271678X16668988 27677673 PMC5435296

[B31] Taha A. Bobi J. Dammers R. Dijkhuizen R. M. Dreyer A. Y. van Es A. C. G. M.. (2022). Comparison of large animal models for acute ischemic stroke: which model to use? Stroke 53, 1411–1422. 10.1161/STROKEAHA.121.036050 35164533 PMC10962757

[B32] Tanaka Y. Imai H. Konno K. Miyagishima T. Kubota C. Puentes S.. (2008). Experimental model of lacunar infarction in the gyrencephalic brain of the miniature pig: neurological assessment and histological, immunohistochemical, and physiological evaluation of dynamic corticospinal tract deformation. Stroke 39, 205–212. 10.1161/STROKEAHA.107.489906 18048856

[B33] Walczak P. Ji X. Li S. Boltze J. (2024). Neurodegeneration in acute and chronic central nervous system disorders: Novel ideas and approaches. Neuroprotection 2, 243–245. 10.1002/nep3.69 40292023 PMC12020453

[B34] Walczak P. Wojtkiewicz J. Nowakowski A. Habich A. Holak P. Xu J.. (2017). Real-time MRI for precise and predictable intra-arterial stem cell delivery to the central nervous system. J. Cereb. Blood Flow Metab. 37, 2346–2358. 10.1177/0271678X16665853 27618834 PMC5531335

[B35] Waseem A. Saudamini Haque R. Janowski M. Raza S. S. (2023). Mesenchymal stem cell-derived exosomes: shaping the next era of stroke treatment. Neuroprotection 1, 99–116. 10.1002/nep3.30 38283953 PMC10811806

[B36] Watanabe H. Sakoh M. Andersen F. Rodell A. Sørensen J. C. Østergaard L.. (2007). Statistical mapping of effects of middle cerebral artery occlusion (MCAO) on blood flow and oxygen consumption in porcine brain. J. Neurosci. Methods 160, 109–115. 10.1016/j.jneumeth.2006.08.016 17129609

[B37] Wright E. A. d'Esterre C. D. Morrison L. B. Cockburn N. Kovacs M. Lee T. Y. (2016). Absolute cerebral blood flow infarction threshold for 3-hour ischemia time determined with CT perfusion and F-18-FFMZ-PET imaging in a porcine model of cerebral ischemia. PLoS ONE 11 10.1371/journal.pone.0158157 27347877 PMC4922566

[B38] Wu D. Chen J. Wu L. Lee H. Shi J. Zhang M.. (2022). A clinically relevant model of focal embolic cerebral ischemia by thrombus and thrombolysis in rhesus monkeys. Nat. Protoc. 17, 2054–2084. 10.1038/s41596-022-00707-5 35760857

[B39] Zhang L. Cheng H. Shi J. Chen J. (2007). Focal epidural cooling reduces the infarction volume of permanent middle cerebral artery occlusion in swine. Surg. Neurol. 67, 117–121. 10.1016/j.surneu.2006.05.064 17254860

[B40] Zhang R. Bertelsen L. B. Flø C. Wang Y. Stødkilde-Jørgensen H. (2016). Establishment and characterization of porcine focal cerebral ischemic model induced by endothelin-1. Neurosci. Lett. 635, 1–7. 10.1016/j.neulet.2016.10.036 27773792

